# CPEB3 functions as a tumor suppressor in colorectal cancer via JAK/STAT signaling

**DOI:** 10.18632/aging.103893

**Published:** 2020-11-03

**Authors:** Yuxin Fang, Qian Zhong, Yadong Wang, Chuncai Gu, Side Liu, Aimin Li, Qun Yan

**Affiliations:** 1 Guangdong Provincial Key Laboratory of Gastroenterology, Department of Gastroenterology, Nanfang Hospital, Southern Medical University, Guangzhou 510515, China

**Keywords:** tumor suppressor, metastasis, proliferation, post-transcriptional regulation, CPEB3

## Abstract

As RNA-binding proteins, cytoplasmic polyadenylation element binding proteins (CPEBs) have drawn increasing attention for their function of controlling gene expression related to malignant transformation via post-transcriptional regulation. However, the contribution of CPEB3 to malignant development in cancers is poorly understood. In this study, we explored the clinical, biological, and mechanical role of CPEB3 in colorectal cancer progression. We showed that colorectal cancer tissues exhibited dampened CPEB3 expression which was closely associated with poor prognosis in patients with colorectal cancer (47 vs. 62 months, *P* = 0.035, n=99). Down-regulation CPEB3 promoted proliferation, migration, and invasion in colorectal cancer cells and vice versa. Mechanistically, CPEB3 performed as an RNA binding protein binding to 3ʹUTR of JAK1 mRNA to inhibit JAK/STAT pathways in colorectal cancer cells. Knockdown of CPEB3 induced active JAK-STAT signaling, thereby triggering the proliferation and metastasis capacity of colorectal cancer cells. These results suggest that CPEB3 functions as a tumor suppressor in colorectal cancer through its post-transcriptional regulation of JAK/STAT signaling. Implications: This study identified a novel role of the RNA binding protein CPEB3 in inhibiting cell proliferation and migration as well as the underlining mechanisms in colorectal cancer cells.

## INTRODUCTION

Colorectal cancer is extremely common and a serious health concern worldwide. Despite improvements in treatment, patients with advanced tumors still suffer from unsatisfactory prognosis [1, 2]. Hence, a better understanding of the molecular mechanisms contributing to colorectal cancer progression is critical for the development of more effective, targeted therapies.

Malignant transformation comprises a complicated process mediated by many pro- and anti-tumorigenic factors involving multiple signal pathways [3]. Although gene reprogramming at the transcriptional level has been widely studied, recent work indicates that mRNA post-transcriptional regulation also constitutes an important step in the regulation of gene expression involved in malignant transformation [4, 5] as well as potentially in the oncogenesis of different cancers. Specifically, cytoplasmic polyadenylation element binding proteins (CPEBs) are RNA-binding proteins that interact with cytoplasmic polyadenylation element (CPE) or U-rich sequences in the 3′ untranslated regions (UTRs) of specific mRNAs to activate or repress translation [6, 7]. The CPEB family consists of four members (CPEB1–4). The functions of CPEB1-mediated translation have been well characterized and include cell cycle [8], cellular senescence [9], and synaptic plasticity [10], but less is known regarding the effects of CPEB2-4 binding to target transcripts.

Decreased CPEB1 expression frequently occurs in ovarian and gastric tumors, as well as in breast, myeloma, and colorectal cancer cell lines. These reduced levels have been associated with malignant cell capacity to promote invasion and vascularization [11, 12]. Moreover, bioinformatics analysis based on 42 microarray studies showed ectopic mRNA expression of CPEB1-4 in 92 human cancers [13]. Notably, *CPEB3* mRNA is consistently down-regulated in digestive tract cancers, suggesting a potential role of CPEB3 in tumorigenesis. However, in contrast to CPEB1, evidence directly linking CPEB3 to malignancies remains limited.

In the present study, we confirmed that CPEB3 expression decreases in colorectal cancer tissues, a pattern that is closely associated with poor patient prognosis. CPEB3 down-regulation promotes colorectal cancer cell proliferation, migration, and invasion *in vitro* and *in vivo*, while up-regulation has an inhibitory effect. Furthermore, CPEB3 associates with tumor-related mRNAs in colorectal cancer cells, specifically targeting the 3ʹUTR of JAK1 mRNA to inhibit JAK/STAT pathways. Active JAK1 translation is induced when CPEB3 is down-regulated, causing abnormal JAK/STAT signaling activation in colorectal cancer. These findings highlight a novel role of CPEB3 post-transcriptional regulation in colorectal cancer and possibly in other digestive tract tumors.

## RESULTS

### CPEB3 is frequently down-regulated in colorectal cancer tissues

**Figure 1:**
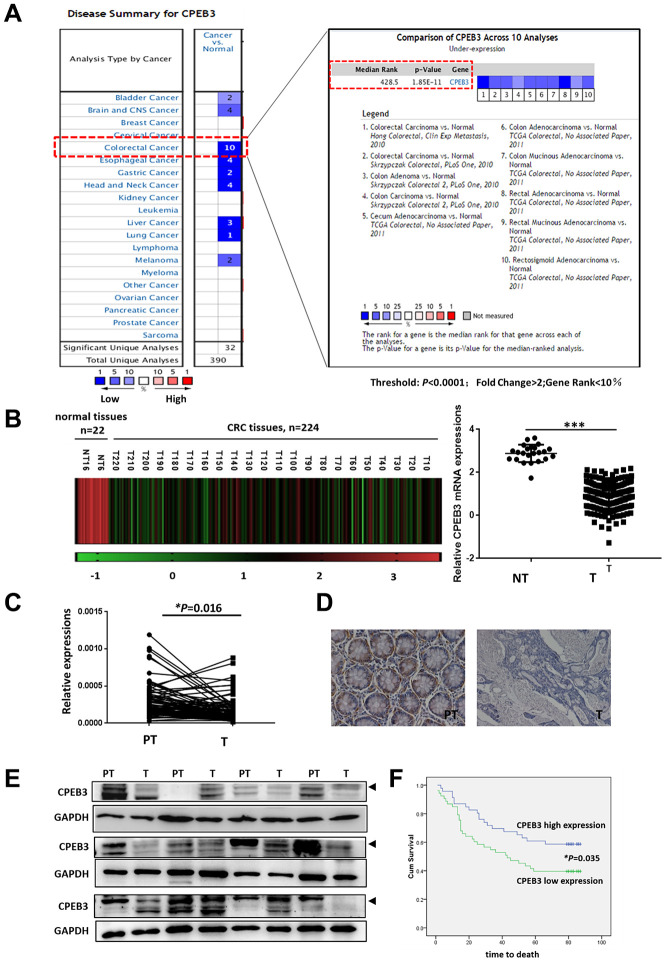
**CPEB3 is frequently down-regulated in colorectal cancer tissues.** (**A**) CPEB3 expression in colorectal cancer tissues and other tumors, based on previously published microarray data from the Oncomine database (threshold: *P* < 0.0001; fold change > 2; gene rank < 10%). (**B**) Analysis of *CPEB3* gene expression in colorectal cancer and normal tissues in TCGA database (n = 246 [224 vs. 22]). (**C**) qPCR analysis of *CPEB3* mRNA expression in human colorectal cancer tissues and matched peri-tumoral tissues (n = 84). (**D**) Representative immunohistochemical staining of CPEB3 in human colorectal cancer tissues (right) and matched peri-tumoral tissues (left). (**E**) Western blotting analysis of CPEB3 in human colorectal cancer tissues and matched peri-tumoral tissues (n = 12). (**F**) Patients in the CPEB3 low-expression group tended to exhibit shorter OS than patients in the high-expression group by Kaplan-Meier analysis (47 vs. 62 months; *P* = 0.035).

Based on previously published microarray data from the Oncomine database, we first analyzed *CPEB3* expression in colorectal cancer tissues and other tumors and identified 10 databases (*P* < 0.0001; Fold Change >2; Gene Rank <10%) for further analysis (Figure 1A). Compared with peri-tumoral tissues, *CPEB3* mRNA expression was significantly decreased in colorectal cancer. The same phenomenon was also identified in other digestive duct tumors, including gastric, esophageal, and liver cancers. Analysis of *CPEB3* expression in The Cancer Gene Atlas revealed a similar declining trend in colorectal cancer tissues (Figure 1B, *P* < 0.0001). We then employed qRT-PCR, IHC, and WB on a panel of human colorectal cancer tissues (n = 84) from patients undergoing surgical resection in our hospital to further confirm CPEB3 ectopic expression. In 64 of 84 cancerous tissue samples, *CPEB3* mRNA expression decreased in relation to adjacent non-tumorous tissues (*P* = 0.016) (Figure 1C). The same outcome was confirmed using IHC and WB (Figure 1D, 1E).

### Decreased CPEB3 expression is associated with poor prognosis in patients with colorectal cancer

To investigate the significance of CPEB3 expression in CRC, CPEB3 expression levels were further correlated with specific clinicopathologic features of 99 patients with CRC on the TMA. As shown in Table 1, we demonstrated the clinical significance of CPEB3 expression by demonstrating its close correlation with lymphatic metastasis (*P* = 0.04), distant metastasis (*P* < 0.01), and AJCC stages (*P* = 0.026). Further Kaplan-Meier analysis revealed that CPEB3 expression correlated significantly with prognosis. Patients in the low-expression group suffered from shorter OS compared with that in high expression group (47 vs. 62 months, *P* = 0.035, Figure 1F).

**Table 1:** Association of CPEB3 expression with clinicopathological characteristics in 99 patients with CRC.

**Variable**	**n**	**CPEB3**		***P***
		**Low (n = 53)**	**High (n = 46)**	
**Age (years)**				
< 70 (45-69)	54	32	22	0.147
≥ 70 (70-91)	45	21	24	
**Gender**				
Male	58	30	28	0.411
Female	41	23	18	
**Pathological grade**				
Well	69	35	34	0.265
Moderate- Poor	30	18	12	
**pT status**				
T1-2	4	1	3	0.257
T3-4	95	52	43	
**pN status**				
N0	52	23	29	*0.040*
N1	47	30	17	
**pM status**				
M0	94	48	46	*0.040*
M1	5	5	*0*	
**Tumor stage**				
I/II	51	22	29	*0.026*
III/IV	48	31	17	
**Tumor size**				
≤ 5 cm	56	32	24	0.268
> 5 cm	43	21	22	
**Tumor number**				
Single	95	49	46	0.078
Multiple	4	4	*0*	

### CPEB3 inhibits human colorectal cancer proliferation *in vitro*

**Figure 2:**
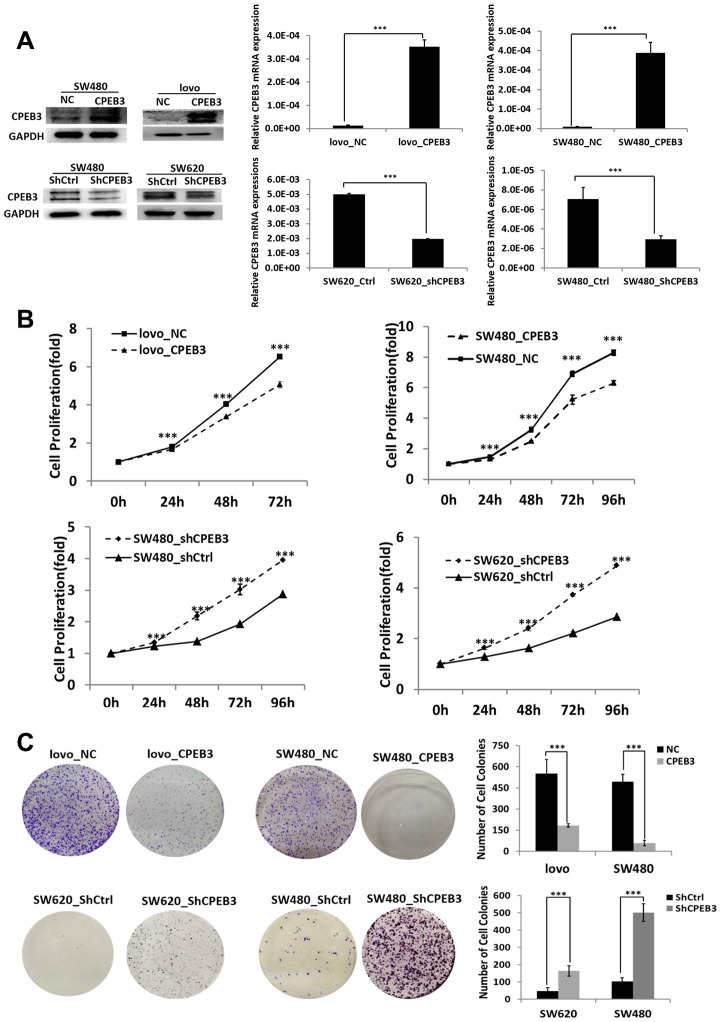
**CPEB3 inhibits human colorectal cancer proliferation *in vitro*.** (**A**) CPEB3 protein expressions of CRC cell lines were efficiently upregulated or downregulated after transfection as analyzed by Western blotting (left) and qPCR (right) detection (**P < 0.01, ***P < 0.001). (**B**, **C**) After effective up- or down-regulation of CPEB3, CCK-8 (**B**) and colony formation (**C**) assays were performed to explore the effect of CPEB3 on proliferative capacity of colorectal cancer cell line.

Our transfection experiments to overexpress or knockdown CPEB3 in three colorectal cancer cell lines were controlled using qPCR and WB experiments (Figure 2A). Based on CCK-8 assay analysis, we found that CPEB3 overexpression significantly suppressed SW480 and LoVo cell proliferation rate at every experimental time point (*P* < 0.001, Figure 2B), whereas CPEB3 knockdown promoted growth rate. Colony formation assay also showed that CPEB3 significantly reduced cancer cell colonies and their size (Figure 2C). Compared with controls, CPEB3 impaired cell cycle progression via inducing G2/M phase arrest in LoVo and SW480 cells (Supplementary Figure 1).

### Overexpression of CPEB3 inhibits a cluster of signaling pathways associated with tumor progression in colorectal cancer cells

**Figure 3:**
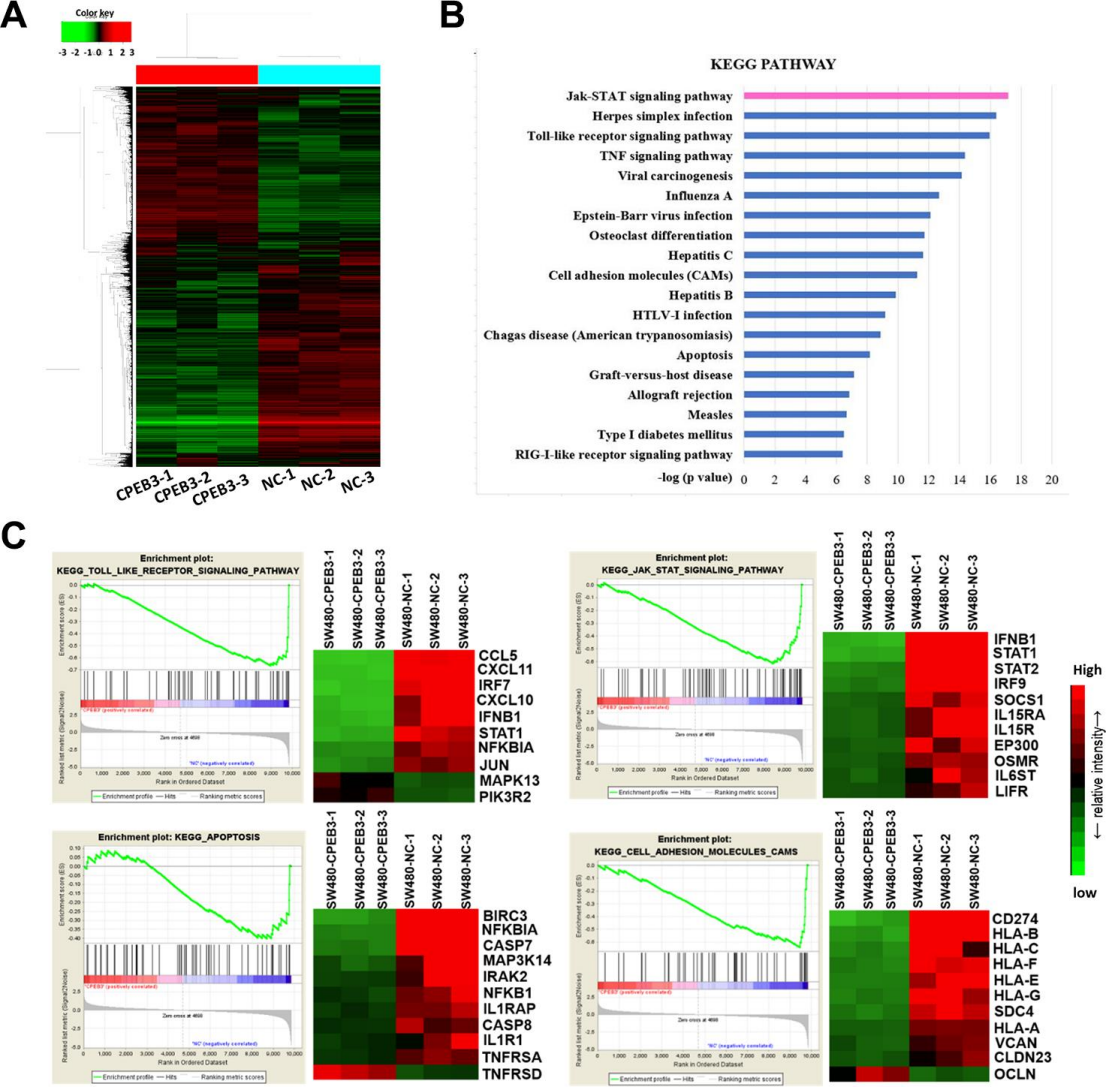
**Overexpression of CPEB3 inhibits a cluster of signaling pathways associated with tumor progression in colorectal cancer cells.** (**A**) Microarray analysis was applied to screen the mRNAs which can be regulated by CPEB3. (**B**) Canonical pathway enrichment of whole genome expression in SW480-CPEB3 and SW480-NC cell lines was analyzed based on KEGG pathway database. (**C**) Gene set enrichment analysis results for all fold changes calculated between SW480-CPEB3 and SW480-NC cells were shown on the left and the heatmap analysis of genes in each signaling pathway were displayed on the right. Red represents high expression levels and green represents low expression levels.

The results of our Illumina whole-genome expression arrays showed that CPEB3 overexpression caused aberrant expression in 1279 genes (362 up-regulated and 917 down-regulated, fold change >1.5 or <0.67 and *P* < 0.05, Figure 3A). According to the analysis of The Cancer Genome Atlas (TCGA) database, overexpression of CPEB3 remarkably suppressed a series of signal pathways including several key carcinoma molecular pathways, such as cell adhesion, apoptosis, JAK/STAT signaling, Toll-Like-receptor signaling and so on (Figure 3B). Gene set enrichment analysis (GSEA) showed a clear stepwise decrease in average gene expression of the four tumor-related pathways from the control group to CPEB3-overexpressed group (Figure 3C). This outcome suggests that CPEB3 might be involved in the regulation of several tumor-related pathways relevant to tumor progression.

### CPEB3 directly targets the 3’UTR of JAK1 and regulates colorectal cancer proliferation and metastasis via CPEB3/JAK/STAT axis

**Figure 4:**
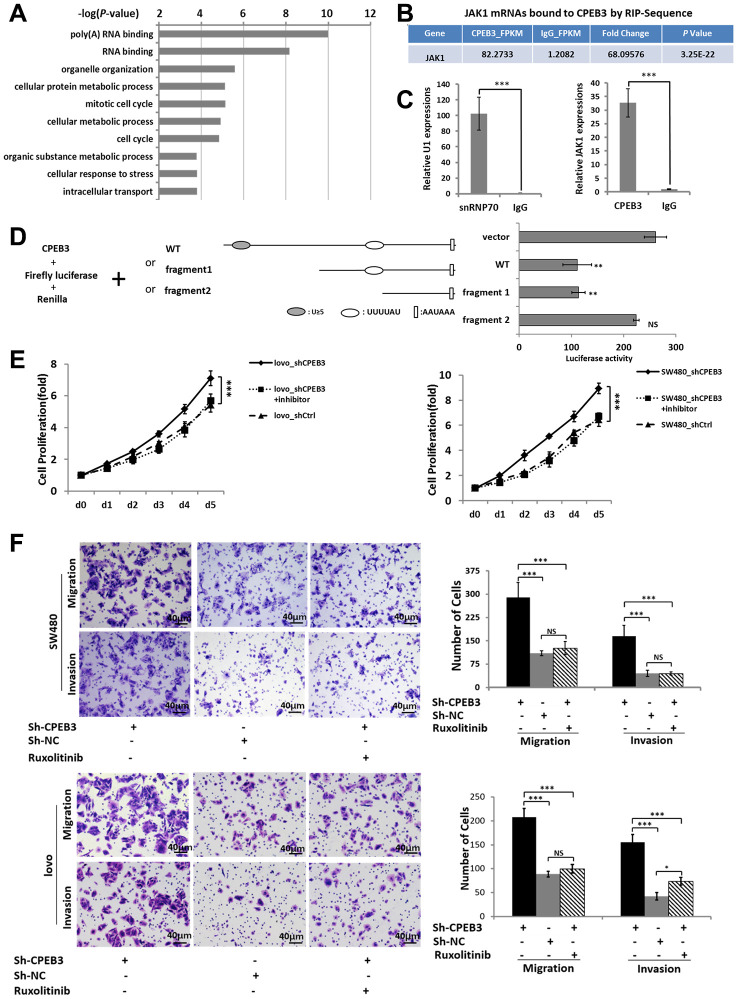
**CPEB3 directly targets the 3’UTR of JAK1 and regulates colorectal cancer proliferation and metastasis via CPEB3/JAK/STAT axis.** (**A**) High-throughput sequencing analysis of mRNAs bound to CPEB3 in SW480 cells showed the top 10 significantly enriched terms using Gene ontology analysis. (**B**) RNA immunoprecipitation and high-throughput sequencing analysis showed that JAK1 mRNA was significantly enriched in CPEB3 immunoprecipitates. (**C**) The combination of JAK1 mRNA and CPEB3 was further validated by RIP-qPCR. IgG and SnRNP70 are used as the negative and positive control respectively. Data are shown as mean ± SD (***P< 0.001). (**D**) Luciferase reporter plasmid appended with different 3’UTR fragments of JAK1 mRNA were co-transfected with CPEB3 in the HEK-293T cell line, and luciferase activity was detected. Data are displayed as the means ± SD (**P< 0.01, NS: No Significance). (**E**, **F**) Impairment of JAK/STAT pathway by Ruxolitinib could reverse the effect of CPEB3 down-regulation on proliferation ability by CCK-8 assay (**E**) as well as the migration and invasion ability by transwell assays (original magnification, ×400) of colorectal cancer cell lines. (**F**).

To obtain a genome-wide picture of potential CPEB3 targets in colorectal cancer, we performed RIP of CPEB3 extracted from SW480 cells. Assay validity was confirmed using snRNP70 (positive control) and IgG (negative control) antibodies for precipitation (Supplementary Figure 2A, 2B). Sequencing analysis of co-immunoprecipitated mRNAs showed that 1791 were significantly (*P* < 1.00 E−05, Fold change > 5) linked to CPEB3 compared with the IgG group (Supplementary Figure 2C). Gene ontology analysis then demonstrated that CPEB3-targeted mRNAs were significantly enriched in biological processes relevant to RNA-binding, cell cycle, cellular response to stress, cellular metabolic process and so on (Figure 4A). We selected JAK1 for further analysis because the transcript was significantly enriched in CPEB3 immunoprecipitates according to the RIP-sequence (Figure 4B) and RIP-qPCR analysis (Figure 4C). Besides, its downstream genes were significantly inhibited following CPEB3 overexpression according to the microarray analysis (Figure 3). The 3’UTR of JAK1 mRNA contains two potential CPEB3 binding sequences including one putative CPE and one U-rich sequence besides the polyadenylation signal (AAUAAA). To determine whether CPEB3 directly binds to JAK1 mRNA, reporter assay with firefly luciferase appended to mRNA of JAK1 3’UTR was performed and the results revealed markedly lower luciferase activity when the full-length sequence of *JAK1* 3′UTR was co-transfected with CPEB3. But this repression was abrogated when the two potential CPEB3 binding sequences was deleted (Figure 4D). Thus, CPEB3 binds to the JAK1 3’UTR.

As we have proved that reduced CPEB3 expression resulted in more aggressive proliferation and metastasis capacity in colorectal cancer cells and CPEB3 involved in the regulation of JAK/STAT signal pathway. We performed CCK-8 and transwell assays to analyze the role of JAK/STAT in CPEB3-mediated cell proliferation and metastasis (Supplementary Figure 3). The results showed that inhibiting JAK/STAT attenuated the stimulatory effects of CPEB3-knockdown on proliferation, migration, and invasion (Figure 4E, 4F). Altogether, these results revealed that there is a mechanistic connection between CPEB3 and the JAK/STAT signal pathway, potentially via direct binding to JAK1 3’UTR in colorectal cancer cells.

### CPEB3 inhibits human colorectal cancer proliferation and JAK/STAT pathway *in vivo*

**Figure 5:**
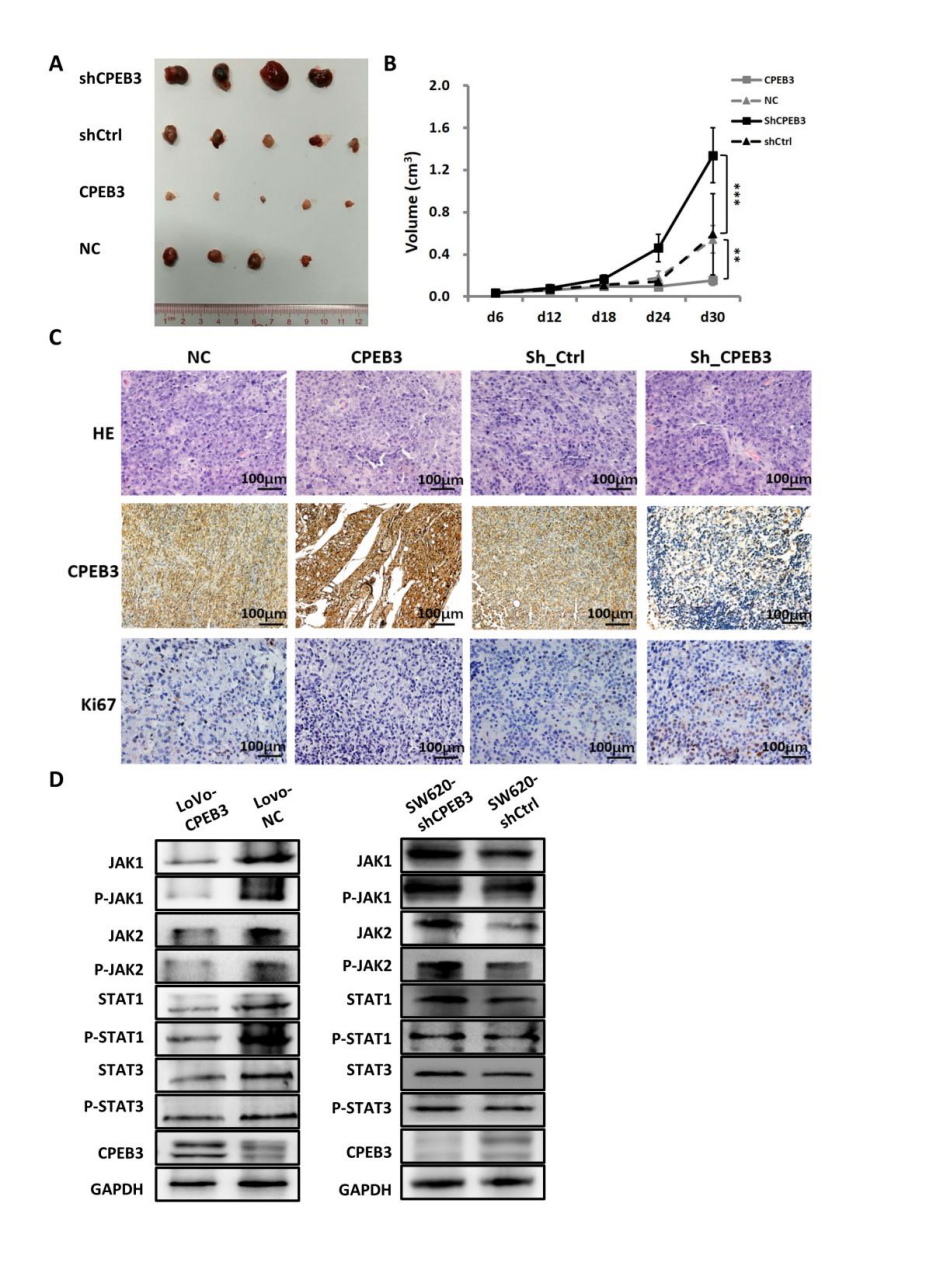
**CPEB3 inhibits human colorectal cancer proliferation and JAK/STAT pathway *in vivo*.** (**A**) Representative images of tumors formed in nude mice subcutaneously implanted with SW480-CPEB3, SW-NC, SW480-shCPEB3 and SW480-shCtrl cell lines. (**B**) Growth curves of implanted tumors in nude mice (**P< 0.01, ***P< 0.001). (**C**) Representative immunohistochemical images of tumor xenografts derived from SW480-CPEB3, SW-NC, SW480-shCPEB3 and SW480-shCtrl cell lines stained by anti-CD31 and anti-Ki67 antibodies (Original magnification, ×200). (**D**) Western blots showed that CPEB3 overexpression suppressed JAK1 and phosphorylated JAK1, as well as molecules in the JAK/STAT pathway while CPEB3 knockdown promoted the activation of these genes.

For the tumorigenicity assay *in vivo*, SW480-CPEB3 and -NC cells as well as SW480-shCPEB3 and SW480-shControl cells were injected subcutaneously in the nude mice. Tumor volume was measured every 6 d and xenograft tumor growth curves were drawn 30 d post-injection. Tumor volume dropped significantly in the CPEB3-overexpressing group compared with control (final volume: 0.155 cm^3^ vs. 0.543 cm^3^, *P* = 0.008), whereas the CPEB3-knockdown group had dramatically larger tumors than control (final volume: 1.338 cm^3^ vs. 0.592 cm^3^, *P* = 0.000) (Figure 5A, 5B). Histopathological detection of xenograft tumors showed that the CPEB3-overexpressing group exhibited weaker Ki67 staining than control, whereas the CPEB3-knockdown group had stronger Ki67 staining (Figure 5C). What’s more, tumors from CPEB3 overexpressed group exhibited decreased activity of JAK/STAT signaling pathway, whereas CPEB3 knockdown promoted their activation (Figure 5D). These results indicated that CPEB3 inhibits colorectal cancer cell proliferation ability and JAK/STAT pathway *in vivo*.

## DISCUSSION

Data accumulated in recent years support the role of post-transcriptional regulation in cancer oncogenesis [14]. RNA-binding proteins (RBPs) are emerging epigenetic post-transcriptional regulators involved in RNA processing, such as splicing, localization, stabilization, and translation. As a consequence, the abundance of RBPs and their constituents differentially affects RNA regulation [15–17]. Interplay between RBPs and RNAs is crucial for normal biological processes (e.g., brain and ovarian development) in addition to regulating tumor progression. CPEBs are RBPs with similar RNA-binding domains, through which they regulate CPE-containing mRNA translation. This capacity means they control the cell cycle, cellular differentiation, or synaptic plasticity.

Initial evidence linking CPEBs to cancer biology emerged from a meta-analysis of 42 studies comparing global gene expression in 92 human cancers with matched normal tissue [13, 18]. The findings strongly support a role of CPEBs in tumorigenesis. First, they showed that CPEB1 mRNA expression is reduced in reproductive-system and brain cancers. Additionally, CPEB4 mRNA is upregulated in pancreatic carcinomas and glioblastomas, while CPEB3 mRNA is consistently downregulated in digestive tract tumors. Subsequently, direct connections between ectopic CPEB1 expression and tumorous development were established [12, 19]. Ovarian and gastric cancer, as well as breast, myeloma, and colorectal cancer cell lines all display dampened CPEB1 expression [11, 12]. Mechanically, CPEB1 interactions with HIF-1a mRNA appear to repress the latter’s translation, thus inhibiting invasion and angiogenesis [20]. What’s more, elevated CPEB4 expression correlates with increased malignancy, tumor growth, and vascularization in pancreatic ductal adenocarcinoma and glioblastoma [21]. However, we know considerably less about CPEB3’s role in cancer development compared with the body of work available for CPEB1 and CPEB4 [13, 22].

In this study, we showed that CPEB3 is frequently down-regulated in colorectal cancer tissues compared with matched peri-tumoral tissues. The clinical significance of CPEB3 expression is demonstrated by close correlations with lymphatic metastasis, distant metastasis, and tumor stage. Lower CPEB3 expression predicts shorter survival time in patients with colorectal cancer after surgical resection. As for the upstream regulatory mechanisms leading to decreased CPEB3 expression in cancers, one recent study by Lin et al. revealed that LncRNA SUMO1P3 promotes proliferation and inhibits apoptosis in colorectal cancer by epigenetically silencing CPEB3 [23]. Another recent study underlined the role of miRNA-301b-3p in the regulation of CPEB3/EGFR axis [24]. These results suggest a potential role of CPEB3 in cancers and highlight post-transcriptional regulation maybe an important controlling mechanism of CPEB3 expression.

In addition, CPEB3 attenuates colorectal cancer cell proliferation, while silencing the protein enhances proliferation rate *in vitro* and in athymic nude mice. Moreover, CPEB3 down-regulation in colorectal cancer cells increased migration and invasion, while up-regulation showed the opposite effect. Together, these results support conclusions from clinical data that CPEB3 is a tumor suppressor in colorectal cancer progression. Consistent with our results, previous research found that CPEB3 represses proliferation and metastasis in hepatocellular carcinoma cells [25]. Moreover, a microarray-based, high throughput screening study for unknown tumor-suppressor genes in colorectal cancer identified CPEB3 as one of four significantly down-regulated genes [26].

The recent development of large-scale quantitative methods, especially next-generation sequencing and modern protein mass spectrometry, facilitates genome-wide identification of RBPs, their protein cofactors, and their RNA targets [27–30]. Notable RBP-immunoprecipitation-based deep-sequencing approaches include crosslinking and immunoprecipitation followed by sequencing (CLIP-seq), RNA immunoprecipitation and sequencing (RIP-seq), as well as *in vitro* evolution methods. Studies using these techniques revealed that many RBPs bind to thousands of RNA targets in cells at defined binding sites [31, 32]. Here, we performed RIP-sequence to describe the RNA profiling directly binding to CPEB3. Consistent with other RBPs, CPEB3 binds to over 1000 RNAs; the most common targets are those encoding translation initiation factors and RNA-binding proteins, supporting the RNA-binding function of CPEBs. In addition, CPEB3 is associated with a gene cluster and signal pathways critical to cancer proliferation and metastasis, such as cellular protein metabolism, cell cycle (cyclins) and cellular stress response (autophagy-related factors, signal transducers and modulators).

Post-RNA-binding mechanisms of CPEB3 also remain poorly understood, especially in tumor development. Previous studies reported that CPEB3 depletion increases glutamate receptor 2 (GLUR2) mRNA translation [33] and CPEB3 inhibits translation of mRNA targets by localizing them to P bodies [34], suggesting that in neurons, CPEB3 has a negative regulatory role in post-transcription. However, another study demonstrated that CPEB3 can stimulate GLUA1 and GLUR2 translation, which suggests a positive regulation of its RNA targets [35]. In this study, whole-genome arrays revealed that CPEB3 overexpression in colorectal cancer cells down-regulated 226 genes by over two-fold, while up-regulating only 30 more. In addition, CPEB3 over-expressing colorectal cancer cells exhibited reduced activity in cancer-related signal pathways, including cell adhesion, apoptosis, JAK/STAT, and Toll-Like-receptor. Therefore, CPEB3 appears to exert primarily a negative effect on gene expression and tumor-related pathways in colorectal cancer cells.

JAKs are mediators of cytokine and growth hormone signaling. Activated JAKs phosphorylate signal transducer and activator of transcription (STAT) proteins, then translocate them from the cytoplasm to the nucleus. There, they bind canonical sequences and modulate transcription of genes involved in cell proliferation, differentiation, and apoptosis [36, 37]. Here, we showed that CPEB3 anti-tumor effects in colorectal cancer are likely mediated via regulating the JAK/STAT pathway. We already know that JAK/STAT signaling pathways are aberrantly activated in several types of cancers, playing a crucial role in promoting carcinogenesis [8, 38, 39]. Here, our microarray analysis showed that CPEB3 overexpression in SW480 cells decreased expression of multiple downstream genes related to the JAK/STST pathway. Our RIP-sequencing and -qPCR also demonstrated that JAK1 is enriched in CPEB3 immunoprecipitates. Furthermore, CPEB3 down-regulation promoted JAK1, P-JAK1, and STAT protein expression, whereas CPEB3 overexpression had the opposite effect. Impairing the JAK/STAT signaling pathway reversed CPEB3-knockdown effects on proliferation, migration, and invasion in colorectal cancer cells. Finally, CPEB3 translationally repressed JAK1 mRNA through binding to 3ʹUTR.

Altogether, this study revealed that colorectal cancer tissues exhibit repressed CPEB3 expression, a phenomenon that predicts poor prognosis in patients with colorectal cancer. In addition, CPEB3 is a tumor suppressor, inhibiting cell proliferation and migration in colorectal cancer cells through regulating the JAK/STAT pathway. However, as few preliminary cancer studies focus on CPEB3, many questions remain unanswered. For example, given the association of CPEB3 with numerous tumor-progression transcripts, we cannot exclude the possibility that other unidentified molecules or compensatory mechanisms may also contribute to aberrant colorectal cancer proliferation and metastasis. Furthermore, data from previously published microarray screening studies have indicated extensive CPEB3 deletion in digestive tract cancers [13]. Thus, the anti-tumorous effects of CPEB3 may not be restricted to colorectal cancer and requires further exploration in other digestive tract cancers.

## MATERIALS AND METHODS

### Patients and tissue samples

84 pairs of tumoral and peri-tumoral tissues for qRT-PCR, Immunohistochemistry (IHC) and Western blotting (WB) analysis were obtained from patients who underwent surgical resection at our institute’s Department of General Surgery. A tissue microarray (TMA) including 99 pairs of CRC tissues with survival data were purchased from the China National Engineering Center for Biochip (Shanghai, China) and IHC detection and survival analysis were performed using the tissue microarray slides. Tumor stage was defined according to the American Joint Committee on Cancer (AJCC)/International Union against Cancer classification system [40]. All procedures were approved by the institutional ethics committee of Nanfang Hospital and all enrolled subjects provided written informed consent.

### Cell culture and lentivirus infection

Human colorectal cancer cell lines LoVo, SW620, SW480 and human embryonic kidney-293T (HEK-293T) were obtained from the Shanghai Institute of Biochemistry and Cell Biology (Shanghai, China) and maintained in Dulbecco’s modified Eagle’s medium (DMEM; Invitrogen, Gaithersburg, MD, USA) containing 10% fetal calf serum (FCS; Hyclone, Logan, UT, USA), 100 IU/mL penicillin, and 100 μg/mL streptomycin. Cell lines were obtained in 2013, authenticated via short tandem repeat fingerprinting, and tested negative for mycoplasma contamination using Hoechst staining and PCR. Lentiviruses carrying full-length *CPEB3* or short hairpin RNA (shRNA) sequences were targeted against human *CPEB3* mRNA and matched negative controls (NC) were constructed by the Shanghai Institute of Biochemistry and Cell Biology. The stable cell lines were established as previously described [41]. To obtain stable transfectants, 5 μg/mL puromycin was added to the culture medium 72 h post-transfection. Cells were pretreated with a dimethylsulfoxide vehicle control or the JAK inhibitor ruxolitinib (3 μM, Selleck Chemicals, Houston, TX, USA) for 48 h prior to analysis.

### RNA extraction, cDNA synthesis, and qRT-PCR

Total RNA was extracted using TRIzol (Invitrogen) for cDNA synthesis using random primers (Supplementary Table 1) and the TaKaRa PrimeScript RT regent kit. Quantitative real-time PCR was performed in triplicate using SYBR Green on a Light Cycler 480 Real Time PCR System (Roche Diagnostics, Mannheim, Germany). Cycling conditions were as follows: 40 cycles of 95°C (30 s), 95°C (5 s), and 60°C (20 s), followed by one cycle of 65°C (15 s). *GAPDH* was used as the endogenous control and relative gene expression levels were calculated using the 2−ΔΔCt method.

### IHC

Tissue sections were deparaffinized in xylene and rehydrated using a graded ethanol series. They were then immersed in 0.1 M citrate (pH 6.0) at 120 °C for 10 min to retrieve antigens, incubated with 1% goat serum albumin to block nonspecific binding, then incubated with CPEB3 polyclonal rabbit anti-human IgG (1:50; Abcam, Cambridge, UK) at 4 °C overnight. Secondary antibody incubation was performed after the sections were kept at room temperature for 45 min. Reactions were developed using diaminobenzidine as a chromogenic substrate. Two observers independently scored sections to determine proportion of positively stained tumor cells (<5% = 0, 5–25% = 1, 26–50% = 2, 51–75% = 3, 76–100% = 4) and staining intensity (none = 0, weak = 1, moderate = 2, strong = 3). The percentage of positive cells of different intensity was multiplied by the corresponding intensity value to obtain the staining index, ranging from 0 to 12. Cutoff values for CPEB3 were chosen based on a measure of heterogeneity by log-rank test statistical analysis with regard to overall survival (OS). An optimal cutoff value was obtained, with scores of>3 and ≤3 defined as the high and low CPEB3 expression group, respectively.

### WB

Cells were homogenized in RIPA lysis buffer (Millipore, Billerica, MA, USA) containing 1% Halt Protease and Phosphatase Inhibitor Cocktails (Thermo Scientific, Rockford, IL, USA) and incubated on ice for 30 min. After centrifugation, proteins in the supernatant were quantified using a Pierce BCA Protein Assay Kit (Thermo Scientific). Equal amounts of proteins were fractionated using sodium dodecyl sulfate-polyacrylamide gel electrophoresis and transferred to polyvinylidene fluoride membranes (Bio-Rad Laboratories, Hercules, CA, USA). Membranes were then blocked with 5% non-fat milk in TBST (10 mM Tris-HCl [pH 8], 150 mM NaCl, 0.05% Tween 20) for 1 h, probed overnight with designated primary antibodies at 4 °C, then incubated with corresponding horseradish peroxidase (HRP)-conjugated secondary antibodies. Signals were developed using Immobilon Western chemiluminescent HRP substrate (Millipore) and scanned in a GeneGnome HR Bioimaging system (Syngene, Bristol, UK). GAPDH was used as an internal control.

### Cell proliferation, colony formation, and cell cycle assay

Colorectal cancer cells (5000 cells/well) were seeded in 96-well plates. Proliferation rate was assessed at 24, 48, 72, and 96 h using the Cell Counting Kit-8 (CCK-8; Beyotime, China). Briefly, cells were incubated with 100 μL DMEM containing 10 μL CCK-8 at 37 °C for 2 h. Absorbance at 450 nm was measured using a microplate spectrometer (Molecular Devices, Sunnyvale, CA, USA). Each time point was assessed in replicates of five wells. For the colony formation assay, stable cell lines (200 cells/well) were seeded in six-well plates. After 2 weeks, cells were fixed in 4% paraformaldehyde and stained with crystal violet for 30 min at room temperature. Colonies consisting of >50 cells were counted. Three independent experiments were performed for each cell line. For cell cycle analysis, stable cell lines were trypsinized, rinsed in PBS, fixed in 70% cold ethanol at 4 °C overnight, and incubated with 0.1 mg/mL RNase A (Calbiochem, San Diego, CA, USA) at 37 °C for 30 min, then with 0.5 mg/mL propidium iodide in the dark at 4 °C for 30 min. Cell cycle distribution was measured using a BD LSRFortessa cell analyzer (BD Biosciences, San Jose, CA, USA) and analyzed using ModFit (Verity Software House, Topsham, ME, USA).

### Migration and invasion assay

The migration and invasion assays were performed in 24-well plates with 8 μm polycarbonate nucleopore filters (3422, Corning, Armonk, NY, USA). The assay membrane was covered with 40 μL BD Matrigel (diluted 1:8 with serum-free medium) in advance. Stable cells lines (1 × 10^5^ and 5 × 10^5^ cells for migration and invasion assays, respectively) were resuspended with 200 μL serum-free medium and seeded in the upper chambers; lower chambers were filled with 500 μL medium containing 10% FCS. After a 48 h incubation, cells adhering to the lower filter surface were fixed with 4% paraformaldehyde and stained with 0.5% crystal violet. Cells in five random fields (at ×200 magnification) were counted under a light microscope. Experiments were independently replicated three times. Data are presented as the means ± SD.

For the wound healing assay, stable cells were seeded into a six-well plate until 80% confluence. Monolayers were scratched with a 200 μL pipette tip to generate wounds. Wells were washed thoroughly with PBS and incubated for another 48 h. Images were taken at 0, 24, and 48 h using a light microscope (×200 magnification). Wound width was measured in Image J (National Institutes of Health, Bethesda, MD, USA) using an average of three width measurements per picture. Experiments were performed in triplicate.

### Tumorigenesis in nude mice

Tumorigenesis testing in nude mice was carried out in accordance with ARRIVE guidelines. Briefly, 4-week-old BALB/c male nude mice were purchased from the Experimental Animal Center of Southern Medical University (Guangzhou, China). Xenograft tumors were generated via subcutaneous injection of SW480 cells (3 × 10^6^) stably expressing CPEB3 or CPEB3-shRNA in the right flank of nude mice. Mice were housed and maintained under specific pathogen-free conditions. Tumor nodules were examined every 6 d to evaluate volume using the following formula: tumor volume = (width^2^ × length)/2. Mice were sacrificed with cervical dislocation 30 d after inoculation; their tumors were excised, weighted, and frozen or fixed for IHC staining and WB examination. Experiments were approved by the Institutional Animal Care and Use Committee of Nanfang Hospital.

### Luciferase reporter assay

HEK-293T cells (3 × 10^4^) were seeded three times independently in 24-well plates. Adhered cells were transfected with 0.5 μg plasmid expressing firefly luciferase reporter appended with human *JAK1*3´UTR mutants, 20 ng plasmid expressing *Renilla* luciferase, and 750 ng plasmid expressing CPEB3 (Obio Technology, Shanghai, China), using Lipofectamine 3000 (Invitrogen). At 24 h after transfection, luciferase activity was measured using the Dual-Luciferase reporter assay system (Promega, Madison, WI, USA).

### Microarray

The GeneChip Human Genome U133 Plus 2.0 Array was used for transcriptome analysis. Briefly, total RNA extracted from SW480-NC and SW480-CPEB3 was purified using RNeasy columns (Qiagen, Valencia, CA, USA). RNA per sample (0.1 μg) was labeled using the MessageAmp Premier RNA Amplification Kit (Ambion, Austin, TX, USA). Biotin-labeled cRNA was obtained from T7-oligo (dT)-primed cDNA processed using the Affymetrix Hybridization, Wash, and Stain Kit, following manufacturer protocol. Finally, hybridized biotinylated cRNA was quantified with a GeneChip Scanner 3000 (Affymetrix).

Image signals were transformed to digital data using Affymetrix GeneChip Command Console. The data were analyzed with Affymetrix GeneChip Command Console Software. Raw data were normalized and perfect matches summarized through median polish using the Robust Multi-array Average algorithm. Differentially expressed genes were analyzed using R Significance Analysis of Microarrays. The significance threshold was q-value <5% and Fold Change >1.5 or <0.67.

### RNA immunoprecipitation and gene sequencing (RIP-seq)

To obtain a genome-wide picture of potential CPEB3 targets in colorectal cancer, we performed RIP of CPEB3 extracted from SW480 cells. SW480 cells grown to 80% confluence were washed with cold PBS and collected via scraping. RNA immunoprecipitation was performed according to Magna RIP RNA-Binding Protein Immunoprecipitation Kit guidelines (Millipore). Briefly, cells were lysed in lysis buffer containing RNase inhibitor and a protease inhibitor cocktail. Magnetic beads were pre-incubated with 5 μg anti-SNRNP70 antibody, anti-CPEB3 antibody, or rabbit IgG for 1 h at room temperature, and lysates were immunoprecipitated with beads at 4 °C overnight. Using RIP Lysate, RNA was extracted from Magna RIP beads for high-throughput sequencing or qRT-PCR detection.

For gene sequencing, RIP-RNAs were subjected to fragmentation, retrotranscription, modification, and PCR to construct the sample library. Cluster generation and sequencing primer hybridization was conducted according to the cBot User Guide. The cluster was processed using a high-throughput sequencing system (HiSeq 2500, Illumina, San Diego, CA, USA) following standard procedures.

For bioinformatics analysis, raw reads (23063600 for *CPEB3* and 21692675 for IgG) were preprocessed by moving the unqualified reads using fastx_tool kit. Approximately 61% of the clean reads for the *CPEB3* group and 33% for the IgG group were rRNAs (Supplementary Table 2). Clean reads were mapped to ensemble HG19 mRNAs using TopHat (version 2.0.9). We obtained 4759549 mapped reads from the *CPEB3* group and 2300735 from the IgG group (Supplementary Table 3). Relative abundances were estimated using cufflinks (version 2.1.1). Fragment per kilobase of transcript per million mapped reads (FPKM) was calculated using the formula: FPKM = total exon fragments / (mapped reads (millions) × exon length (kb)). Genes with significant differences were selected based on the threshold criteria of *P* < 0.0001 (10^−4^) and fold changes >5.

### Statistical analysis

All statistical analyses were performed in SPSS version 19.0 (Chicago, IL, USA). Pearson's chi-square was used to analyze the relationship between CPEB3 expression and the clinicopathological features of patients with colorectal cancer. Overall survival rates were calculated using the Kaplan-Meier method, and significance was determined using a log-rank test. Paired-sample t-tests were used to determine differences between cancerous and peri-tumoral tissues. All other between-group differences were analyzed with two-tailed independent Student’s t-tests. Data are presented as the means ± SD. Significance was set to *P* < 0.05.

## Supplementary Material

Supplementary Figures

Supplementary Tables
