# Supplementary material for: CPEB3 functions as a tumor suppressor in colorectal cancer via JAK/STAT signaling
Source: Aging (Albany NY). 2020 Nov 3;12(21):21404–22. doi: 10.18632/aging.103893 (PMC7695424; doi:10.18632/aging.103893)
Supplement: Supplementary Figures [file aging-12-103893-s001..pdf]

SUPPLEMENTARY FIGURES

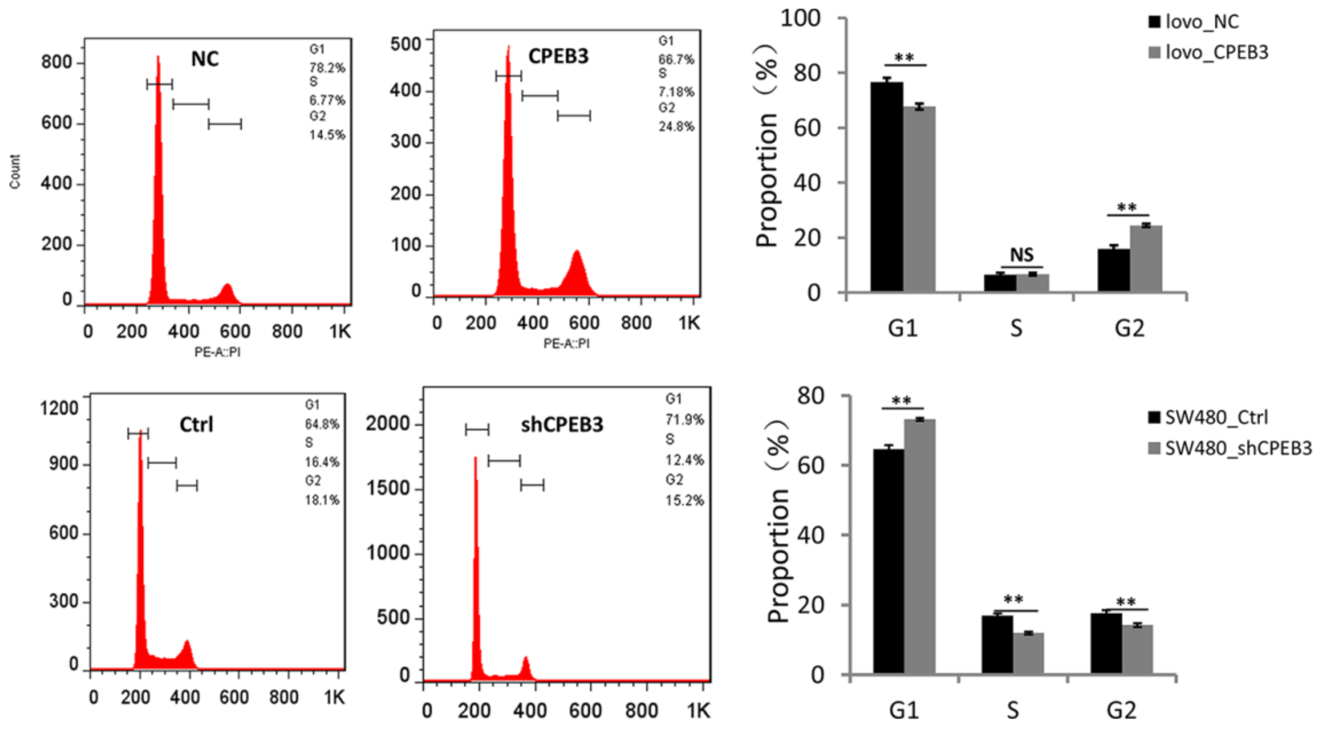

Supplementary Figure 1. Up-regulation of CPEB3 in LoVo cells results in inhibition of cell cycle progression in G2/M phase as detected by flow cytometry.

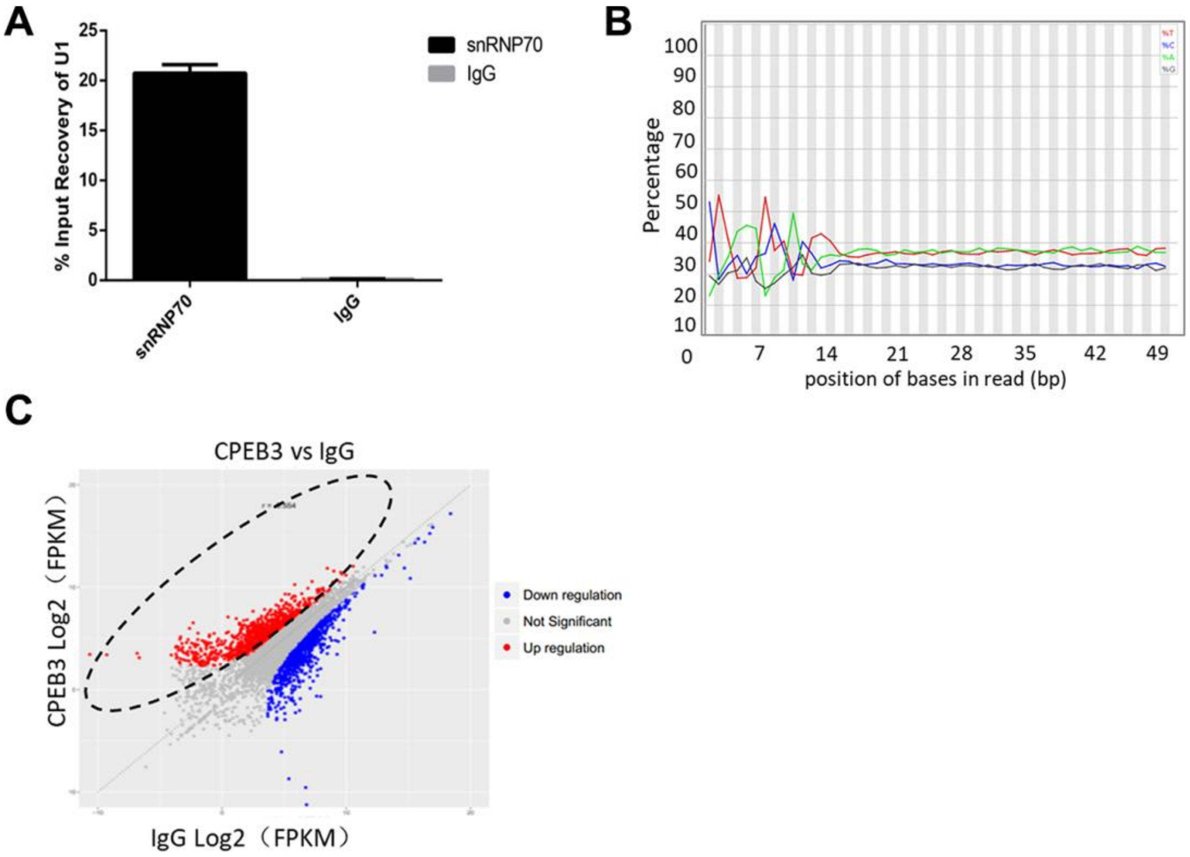

Supplementary Figure 2. High-throughput sequencing analysis of mRNAs bound to CPEB3 in SW480 cells. (**A**) RNA extracts pre-incubated with IgG and SnRNP70 were used as negative and positive controls, respectively. (**B**) Sequence content across all in CPEB3 group. X axis represents the position of bases in reads (bp) and Y-axis represents the percentage of the bases in each position. (**C**) Scatter-plot of the read-densities for the mRNAs in the CPEB3 and the IgG group.

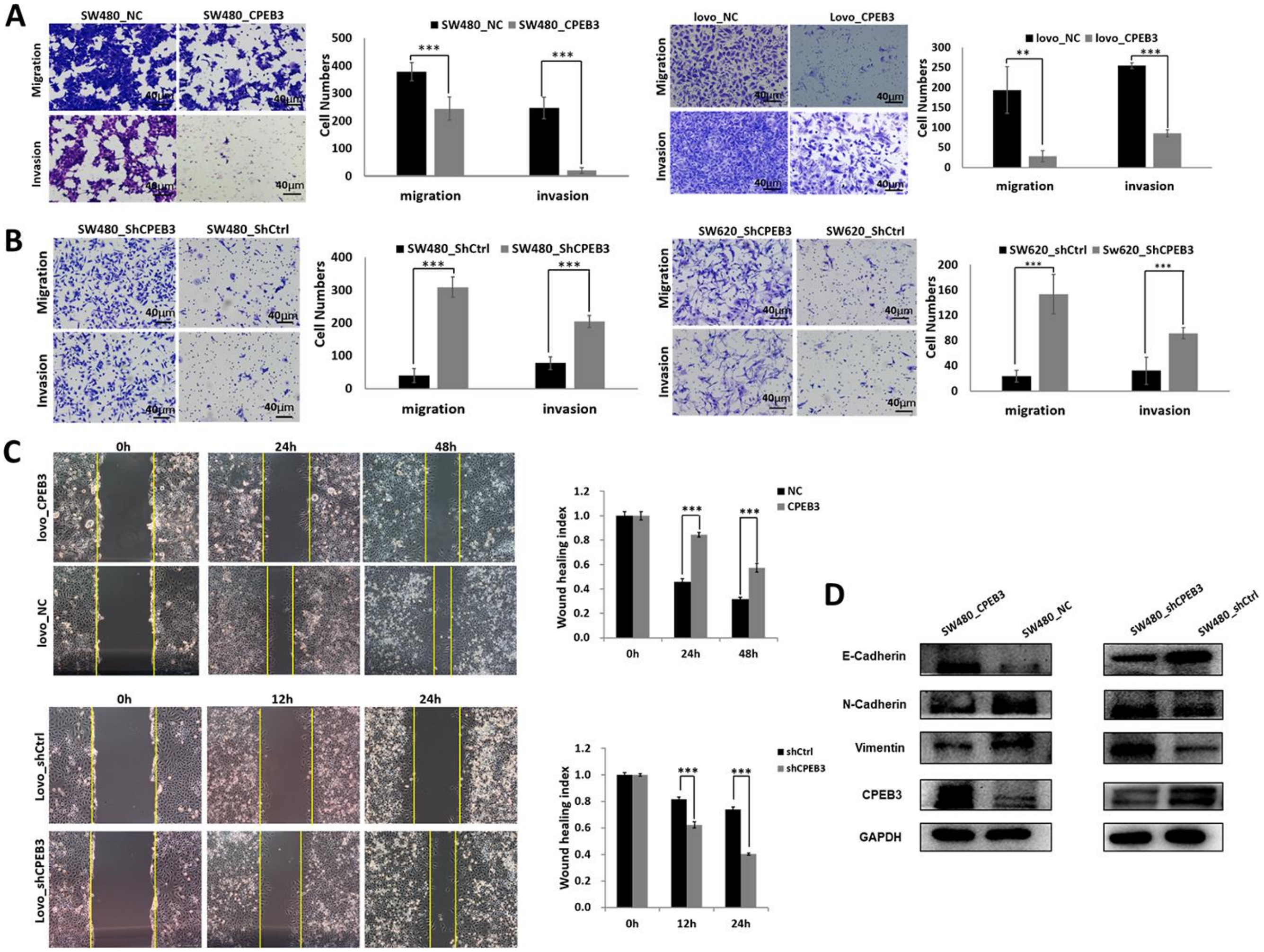

Supplementary Figure 3. CPEB3 inhibits metastasis of human colorectal cancer cells. (**A**) CPEB3 overexpression significantly suppressed migration and invasion capacity of SW480 and SW620 cells by transwell assays (original magnification, ×400). (**B**) CPEB3 knockdown promoted migration and invasion ability of SW480 and SW620 cells (original magnification, ×400). (**C**) In vitro wound healing assays showed that CPEB3 suppressed the migration ability of LoVo cells (original magnification, ×100). (**P< 0.01, ***P< 0.001). (**D**) Metastasis-related markers were further confirmed by Western blotting.
