# Supplementary material for: CPEB3 functions as a tumor suppressor in colorectal cancer via JAK/STAT signaling
Source: Aging (Albany NY). 2020 Nov 3;12(21):21404–22. doi: 10.18632/aging.103893 (PMC7695424; doi:10.18632/aging.103893)
Supplement: Supplementary Tables [file aging-12-103893-s002..pdf]

SUPPLEMENTARY TABLES

Supplementary Table 1. The upstream and downstream sequence of primers in Real time PCR.

Primer	Primer sequences	
	Forward	Reverse
CPEB3	5-CCAACCTGAGTCCAGCGTAT-3	5-AGGAGCGGTGATTCCATCT-3
MYC	5-GCCCCTCAACGTTAGCTTCA-3	5-AGTTCTCCTCCTCGTCGCAG-3
TGFB1	5-GATGTCACCGGAGTTGTGC-3	5-GAACCCGTTGATGTCCACTT-3
JAK1	5-ACTGAGGTGAACCTGGAGGC-3	5-ACTGCCCAGCCGGAGG-3
SMAD2	5-GGAATTTGCTGCTCTTCTGG-3	5-CTGCCTTCGGTATTCTGCTC-3
STAT3	5-GAGCTGGCTGACTGGAAGAG-3	5-TGTTGACGGGTCTGAAGTTG-3
ATG3	5-TGCTATAAGCGGTGCAAACA-3	5-CGGCTTCCGTTATTCCTGTA-3
STAT6	5-CACTGGAAGCAGGAAGAACTCA-3	5-AGACACTTGGCCAGCCTCA-3
PIK3C3	5-TCATGGACAAGCTGTTACGG-3	5-TCAGCCACAGGAACTGACTG-3
RB1	5-CACATTCCTCGAAGCCCTTA-3	5-GTTGGTGTTGGCAGACCTTC-3
E2F3	5-TGCAGTCTGTCTGAGGATGG-3	5-GGTGCAGCTTTGGATCAGTT-3
GAPDH	5-GAAGGTGAAGGTCGGAGT-3	5-GAAGATGGTGATGGGATTTC-3

Supplementary Table 2. Read count for sample (CPEB3) and control (IgG) in total, and in ribosomal RNAs (rRNAs).

Sample ID	Raw reads	Clean reads	Clean ratio*	rRNA trimed	rRNA ratio**
CPEB3	23063600	22537635	97.72%	8656239	61.59%
IgG	21692675	18560899	85.56%	12404800	33.17%

*: Clean ratio=(clean reads/Raw reads)%; **:rRNA ratio=[(Clean reads-rRNA trimed)/Clean reads]

Supplementary Table 3. Percentage of mapped reads in CPEB3 and control IgG group.

Sample ID	All reads	Mapped reads	Mapped ratio*
CPEB3	8656239	4759549	54.89%
IgG	12404800	2300735	18.55%

*: Mapped ratio=(Mapped reads/All reads)%
